# Identification of microsatellite instability and immune-related prognostic biomarkers in colon adenocarcinoma

**DOI:** 10.3389/fimmu.2022.988303

**Published:** 2022-10-07

**Authors:** Ziquan Sun, Guodong Li, Desi Shang, Jinning Zhang, Lianjie Ai, Ming Liu

**Affiliations:** ^1^ Colorectal Cancer Surgery Department, The Second Affiliated Hospital of Harbin Medical University, Harbin, China; ^2^ Department of General Surgery, The Fourth Affiliated Hospital of Harbin Medical University, Harbin, China; ^3^ College of Bioinformatics Science and Technology, Harbin Medical University, Harbin, China

**Keywords:** colon adenocarcinoma (COAD), microsatellite instability (MSI), immune microenvironment, prognostic biomarkers, drug response

## Abstract

**Background:**

Colon adenocarcinoma (COAD) is a prevalent malignancy that causes significant mortality. Microsatellite instability plays a pivotal function in COAD development and immunotherapy resistance. However, the detailed underlying mechanism requires further investigation. Consequently, identifying molecular biomarkers with prognostic significance and revealing the role of MSI in COAD is important for addressing key obstacles in the available treatments.

**Methods:**

CIBERSORT and ESTIMATE analyses were performed to evaluate immune infiltration in COAD samples, followed by correlation analysis for MSI and immune infiltration. Then, differentially expressed genes (DEGs) in MSI and microsatellite stability (MSS) samples were identified and subjected to weighted gene co-expression network analysis (WGCNA). A prognostic model was established with univariate cox regression and LASSO analyses, then evaluated with Kaplan-Meier analysis. The correlation between the prognostic model and immune checkpoint inhibitor (ICI) response was also analyzed.

**Results:**

In total, 701 significant DEGs related to MSI status were identified, and WGCNA revealed two modules associated with the immune score. Then, a seven-gene prognostic model was constructed using LASSO and univariate cox regression analyses to predict survival and ICI response. The high-risk score patients in TCGA and GEO cohorts presented a poor prognosis, as well as a high immune checkpoint expression, so they are more likely to benefit from ICI treatment.

**Conclusion:**

The seven-gene prognostic model constructed could predict the survival of COAD and ICI response and serve as a reference for immunotherapy decisions.

## Introduction

Colorectal cancer (CRC) is among the most prevalent cancers globally and is ranked the second most common cause of cancer-related death (1). In developed countries, CRC patients’ 5-year survival has been enhanced by early detection, yet 25% present with stage four and additional 25%–50% present in the early stages but progress to metastasis (2). Therefore, further research for effective treatment development is urgently required. Over the past decade, immunotherapy has achieved long-term durable effective responses in treating tumors, including lung cancer and melanoma (2). For CRC, immune checkpoint therapy was approved in 2017 for treating tumors with heavy mutations that have mismatch-repair-deficiency (dMMR) or high levels of microsatellite instability (MSI-H), also known as dMMR-MSI-H tumors. Pembrolizumab obtained FDA approval for treating solid tumors with MSI-Hor dMMR (2).

Colon cancer can be categorized into mismatch-repair-proficient (pMMR), microsatellite stability (MSS), and dMMR microsatellite instability (MSI) subtypes (3). Recent studies have revealed that CRC patients who benefit from immune checkpoint inhibitors mainly have a high mutation burden and mismatch repair deficiency (MSI) (4). In several tumors, the immune cell infiltration biological characteristics and prognostic value have been thoroughly described (3), but the value of MSI as a biomarker remains limited. For example, several clinical trials revealed that metastatic CRC (mCRC) patients with MMR deficiency/MSI-H benefit from the immune checkpoint inhibitor (ICI) treatment (5). However, the efficacy of MSI for drug response and treatment benefit prediction of patients with COAD is unclear. Chen T et al. also developed a lncRNA model to predict gastric cancer’s MSI and prognosis (6). Hence, exploring MSI application in COAD therapy and biomarker identification is necessary. This requires identifying accurate predictive biomarkers to comprehend the pathogenesis, predict the clinical outcomes, and subsequently develop a treatment plan for COAD patients.

The tumor microenvironment (TME) and cancer evolution are strongly co-dependent (7, 8). TME comprises several cellular components, such as endothelial cells, fibroblasts, lymph vessels, blood vessels, and immune cells (9). The immune microenvironment has a crucial function in cancer development and therapy, as the immune system components are usually affected by cancers (10–12). Due to the heterogeneity and complexity of tumor immune microenvironment, few patients have benefited from immunotherapy (13), leading to diverse immunotherapy effects among COAD patients (14). The MSI status alone cannot predict the immune checkpoint blockade therapy response because of the complicated interaction between tumor and immune cells (15). Besides, COAD patients’ prognosis could be predicted by immune-related parameters (14, 16). Consequently, the immune-related and MSI status for identifying prognosis biomarkers is necessary.

This study used MSI and immune-related gene modules to construct and evaluate a prognostic model. Moreover, the prognostic model and drug sensitivity correlation were analyzed using drug response datasets.

## Methods and materials

### Colon adenocarcinoma datasets acquisition

The UCSC Xena (https://xenabrowser.net/) was utilized to obtain clinical and gene expression data of samples from COAD patients in the Cancer Genome Atlas (TCGA). MSI or microsatellite stability (MSS) of TCGA COAD samples was obtained from Zaravinos et al. (17). In TCGA cohort, the clinical-pathological stage and microsatellite status were evaluated using the chi-square test and considered statistically significant if the P-value was less than 0.05 (**Table 1**).

**Table 1:** Baseline characteristics of patients in TCGA COAD cohort.

Characteristics	Whole Cohort	MSI Group	MSS Group	*P*
TCGA cohort	(n=432)	(n=157)	(n=275)	
Gender				0.0034
Male	230 (53.24%)	69 (43.95%)	161 (58.55%)	
Female	202 (46.76%)	88 (56.05%)	114 (41.45%)	
Age				0.17
<65 years	164 (37.96%)	53 (33.76%)	111 (40.36%)	
>=65 years	268 (62.04%)	104 (66.24%)	164 (59.64%)	
T-stage				1
T1	11 (2.55%)	4 (2.55%)	7 (2.55%)	
T2	73 (16.9%)	26 (16.56%)	47 (17.09%)	
T3	293 (67.82%)	107 (68.15%)	186 (67.64%)	
T4	54 (12.5%)	20 (12.74%)	34 (12.36%)	
N-stage				0.012
N0	250 (57.87%)	105 (66.88%)	145 (52.73%)	
N1	103 (23.84%)	32 (20.38%)	71 (25.82%)	
N2	79 (18.29%)	20 (12.74%)	59 (21.45%)	
M-stage				0.049
M0	314 (72.69%)	118 (75.16%)	196 (71.27%)	
M1	64 (14.81%)	17 (10.83%)	47 (17.09%)	
Stage				0.0079
I	71 (16.44%)	27 (17.2%)	44 (16%)	
II	150 (34.72%)	69 (43.95%)	81 (29.45%)	
III	122 (28.24%)	35 (22.29%)	87 (31.64%)	
IV	64 (14.81%)	17 (10.83%)	47 (17.09%)	

Expression levels were detected using a microarray of two datasets with corresponding clinical information (GSE17536 and GSE39582), four datasets with corresponding microsatellite stability status (GSE13294, GSE18088, GSE13067, and GSE72969), and gene expression of two datasets (GSE33113 and GSE17537) were obtained using Gene Expression Omnibus (GEO) (http://www.ncbi.nlm.nih.gov/geo), serving as the validation sets.

### Evaluation of the correlation between microsatellite stability status and tumor immune infiltration

Depending on CIBERSORT, the number of each tumor-infiltrating immune cell type was determined (18). CIBERSORT is a tool that estimates specific types of cell abundance based on the gene expression in a mixed cell population, and mRNA expression data were used in this study to compute the range of 22 infiltrating immune cells in TCGA cohort. CIBERSORT score is available on their website (https://cibersort.stanford.edu/index.php) with 1000 permutations. Additionally, the tumor purity score, the stromal cell level, and the level of infiltrated immune cells in TCGA COAD tumor tissues were determined according to ESTIMATE (Estimation of STromal and Immune cells in MAlignant Tumor tissues) method *via* the “estimate” R package (19).

The expression data of five immune checkpoints were extracted from TCGA cohort; CD274 (code PD-L1), PDCD1 (code PD-1), BTLA, CD47, and CTLA4. A one-sided Wilcoxon rank-sum test was employed for evaluating differences in CIBERSORT 22 immune cells score, ESTIMATE score, and five immune checkpoints expression between the MSI and MSS groups or MSI-H and microsatellite instability low (MSI-L) groups. A P-value less than 0.05 was considered significant.

### Differentially expressed genes and functional analysis

TCGA cohort gene expression data were standardized before performing a differential expression analysis using “edgeR” R package for DEGs detection in MSI and MSS samples using a threshold of FDR < 0.05 and |logFC| > 1. In total, 701 DEGs were identified (**Table S1**), and those DEGs with GO Biological Processes were analyzed using the pathway and process enrichment analysis using Metascape web-based tool (https://metascape.org/gp/index.html) with default settings: terms with an enrichment factor > 1.5, a minimum count of 3, and P < 0.01. The Metascape data are always up to date.

### Weighted gene co-expression network analysis (WGCNA) to identify immune-related modules

WGCNA is a data reduction and unsupervised classification method (20, 21). Subsequently, depending on DEG expression profile, the co-expression network was built using “WGCNA” R package with a parameters set as follows: mergeCutHeight = 0.25, minModuleSize = 20, corType = “Pearson”. The module-trait association method was used to determine the co-expression module related to immune infiltration without impact on the clinical characteristics (**Table S2**). After gene clustering, the modules and phenotype correlation were illustrated by a heatmap. The blue and turquoise modules were eligibly selected.

### Construction of a prognostic model

In TCGA cohort, univariate Cox proportional regression was conducted on “blue” as well as “turquoise” module genes linked to OS. Seventy-eight genes with a P value of less than 0.01 were considered for further analysis. In Cox regression model, the considerable prognostic genes were identified by the least absolute shrinkage and selection operator (LASSO) method for variable selection, as well as one standard error (SE) above minimum criteria. The following risk score formula is presented: Risk score = (exp Gene1 * coef Gene1) + (exp Gene2 * coef Gene2) + … +(exp Gene7* coef Gene7), considering the optimized gene expression and the correlation estimated Cox regression coefficients. COAD patients were categorized into two risk groups according to the given risk score median, and their survival time differences were evaluated using a log-rank test. The findings were presented using Kaplan-Meier plots. The risk score differences between MSI and MSS groups or MSI-H and MSI-L groups in TCGA, GSE13294, GSE18088, and GSE13067 cohorts were evaluated using a one-sided Wilcoxon rank-sum and demonstrated statistical significance when the p-value < 0.05.

### The risk score and drug response correlation analysis

IMvigor210 was a single-arm phase 2 study to investigate atezolizumab in metastatic urothelial cancer (mUCC) patients (NCT02108652, NCT02951767) (22). The IMvigor210 trial complete expression and clinical data were obtained using “IMvigor210CoreBiologies” R package obtained from http://research-pub.gene.com/IMvigor210CoreBiologies. The risk score difference between the drug response (PD [progressive disease], PR [partial response], SD [stable disease], and CR [complete response]) was assessed. The difference in mutation and neoantigen burdens between the risk groups was evaluated by a one-sided Wilcoxon rank-sum. Differential expression for five immune checkpoints between the two risk groups was evaluated in IMvigor210 and TCGA cohorts. The Genomics of Drug Sensitivity in Cancer (GDSC, http://www.cancerrxgene.org/) was utilized to obtain the drug response measurements as LN_IC50 (natural log of the fitted half-maximal inhibitory concentration) and transcription profiles for about 1000 cancer cell lines and drugs targets/pathways. The drug sensitivity and risk score correlation were calculated using Pearson correlation analysis.

### Immunohistochemical verification

Twenty colorectal cancer tissues, including 10 MSI and 10 MSS, were acquired from the Fourth Affiliated Hospital of Harbin Medical University. Immunohistochemistry was performed as previously described (23). Tissues were incubated with anti-CALB2 (ABclonal, dilution 1:100) antibody at 37°C for 1h and with secondary antibodies at room temperature for 30 min. The Olympus BX53 microscope was utilized to capture images, and the immunohistochemical integral optical density (IOD) was analyzed using Image-Pro Plus v6.0. The groups’ average optical densities were compared. The groups’ average optical densities were compared.

### Statistical analysis

Statistical analysis was conducted using GraphPad Prism 8 and R 3.6.3 (https://www.r-project.org/). For comparing the continuous variables in immunohistochemical analysis, the t-test was employed. We applied the Kruskal-Wallis test to compare the continuous variables during the bioinformatic analysis. The subgroups were divided based on the median value. Kaplan-Meier survival analysis was used to generate overall survival curves, and the log-rank test was used to calculate the significance.

## Results

### Microsatellite stability status affected tumor immune infiltration

**Figure 1:**
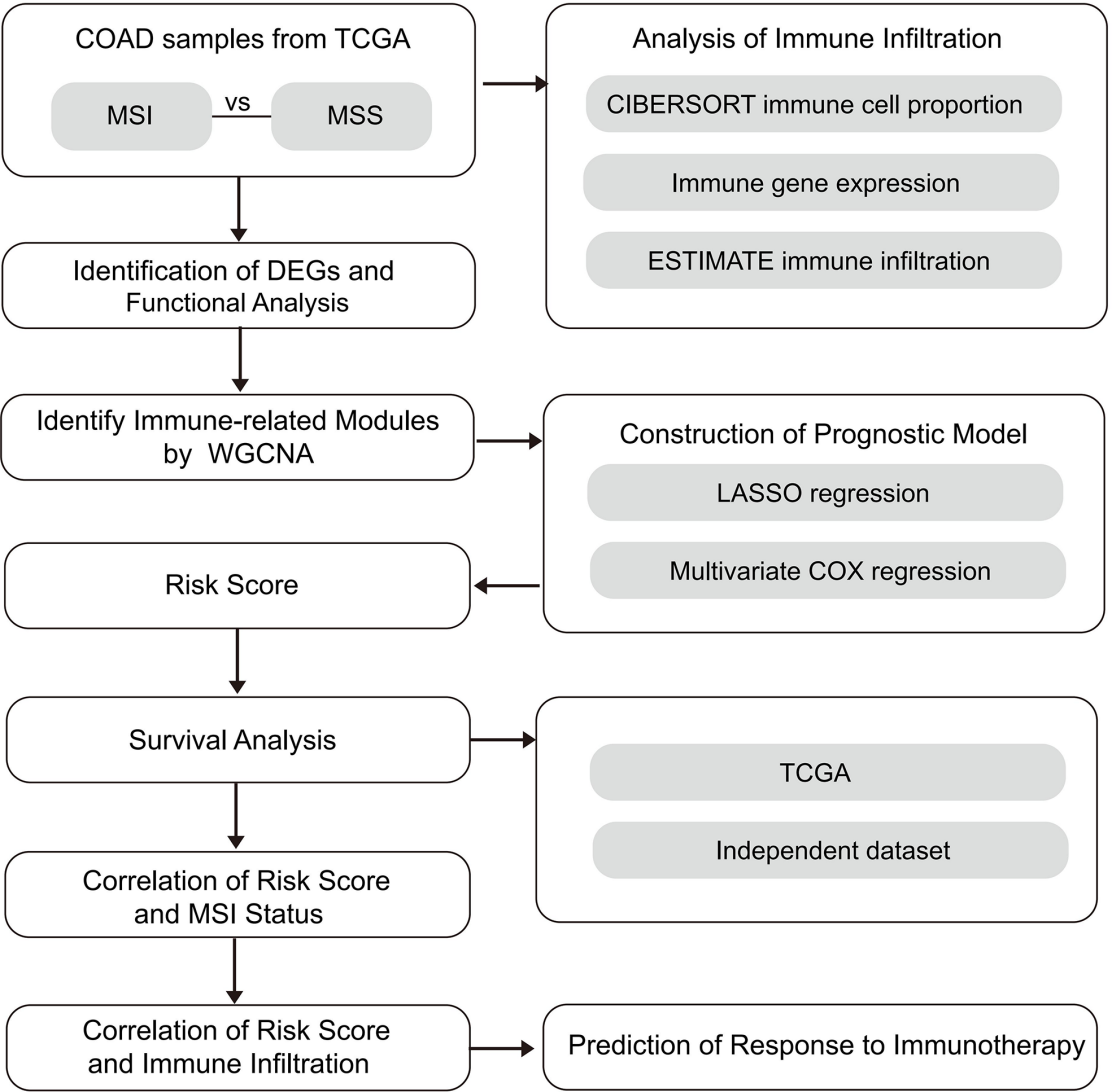
The study design schematic diagram.

**Figure 2:**
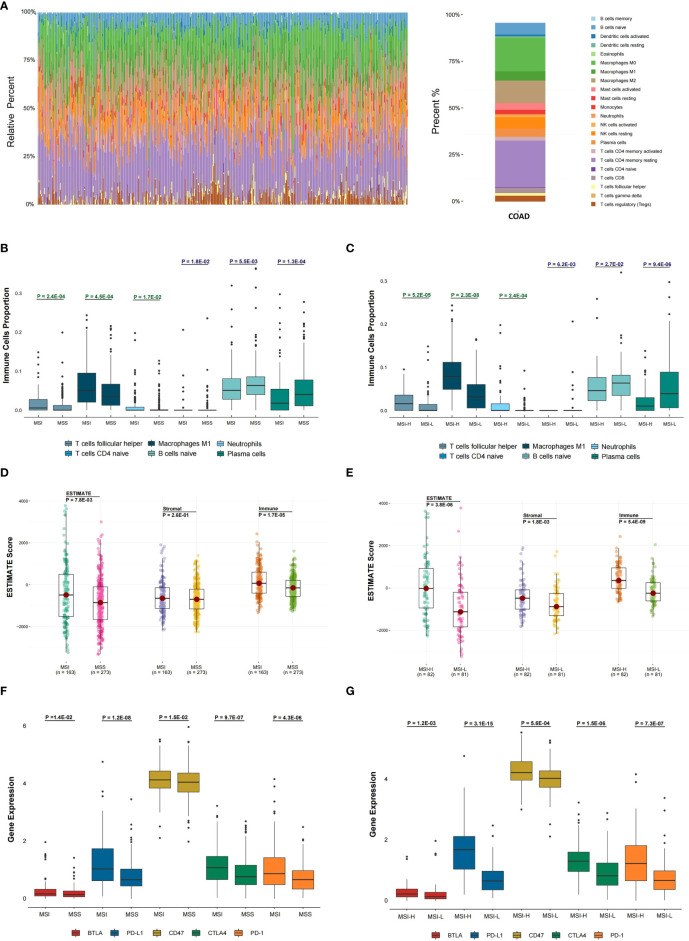
Evaluation of the association between microsatellite stability status and tumor immune infiltration. **(A)** In the TCGA COAD cohort, 22 immune cell proportion and distribution using CIBERSORT are shown. **(B)** The six immune cells infiltration difference between MSI and MSS groups. **(C)** The difference of six immune cells infiltration between MSI-L and MSI-H groups and **(D)** The difference in ESTIMATE score between MSI and MSS groups was analyzed. **(E)** The difference in ESTIMATE score between MSI-L and MSI-H groups was analyzed. **(F, G)** The differential expression status of five immune checkpoints between different MSI groups was analyzed. The one-sided Wilcoxon rank-sum test was utilized to compute P-values.

The abundance of twenty-two immune cells within TCGA COAD samples was calculated by CIBERSORT to evaluate the immune cell infiltration (**Figure 1**). Immune profile for the evaluation of the immune cell infiltration was shown in **Table S3**. Then, the proportions of different subpopulations of tumor-infiltrating immune cells were explored in TCGA COAD (**Figure 2A**). “CD4 memory resting T cells” and “M0 macrophages” represent a significant proportion of COAD immune cell infiltration. Next, we assessed the differentially infiltrated immune cells between MSI and MSS groups (**Figure 2B**), with the infiltration of “follicular helper T cells” (P = 2.4E-04), “M1 macrophages” (P = 4.5E-04), and “neutrophils” (P = 1.7E-02) in MSI group higher than in MSS group, and infiltration of “CD4 naive T cells” (P = 1.8E-02), “naive B cells” (P = 5.5E-03) and “plasma cells” (P = 1.3E-04) in MSI group lower than in MSS group. Besides, we assessed the immune cell infiltration in MSI-H and MSI-L groups (**Figure 2C**), showing that the infiltration of “follicular helper T cells” (P = 5.2E-05), “M1 macrophages” (P = 2.3E-08), and “neutrophils” (P = 2.4E-04) in MSI-L group was lower than in MSI-H group, and the infiltration of “CD4 naive T cells” (P = 6.2E-03), “naive B cells” (P = 2.7E-02) and “plasma cells” (P = 9.4E-06) in MSI-H group was lower compared to MSI-L group. Besides, infiltration of CD8 T cells in MSI group was considerably higher than in MSS group (**Figure S1A**; P = 1.8E-02), and infiltration in MSI-L group was lower than in MSI-H group (**Figure S1B**; P = 8.8E-02).

To explore the tumor purity distinction between different microsatellite stability statuses in TCGA COAD tumor tissues, the ESTIMATE method was applied to evaluate the level of stromal cells and the immune cell infiltration, and these are the basis for ESTIMATE score. The ESTIMATE score (P = 7.8E-03), Stromal score (P = 2.6E-01) and Immune score (P = 1.7E-05) in MSI group were higher than MSS group (**Figure 2D**). The ESTIMATE score (P = 3.8E-06), Stromal score (P = 1.8E-03) and Immune score (P = 5.4E-09) in MSI-L group were lower than in MSI-H group (**Figure 2E**).

The differential expression of five immune checkpoints in the microsatellite instability groups were then analyzed, showing that expressions of BTLA (P = 1.4E-02), PD-L1 (P = 1.2E-08), CD47 (P = 1.5E-02), CTLA-4 (P = 9.7E-07) and PD-1 (P = 4.3E-06) in MSI group were higher than in MSS group (**Figure 2F**), with BTLA expression (P = 1.2E-03), PD-L1 (P = 3.1E-15), CD47 (P = 5.6E-04), CTLA-4 (P = 1.5E-06) and PD-1(P = 7.3E-07) in MSI-L group lower than in MSI-H group (**Figure 2G**).

### Identifying differentially expressed genes between different microsatellite stability status

**Figure 3:**
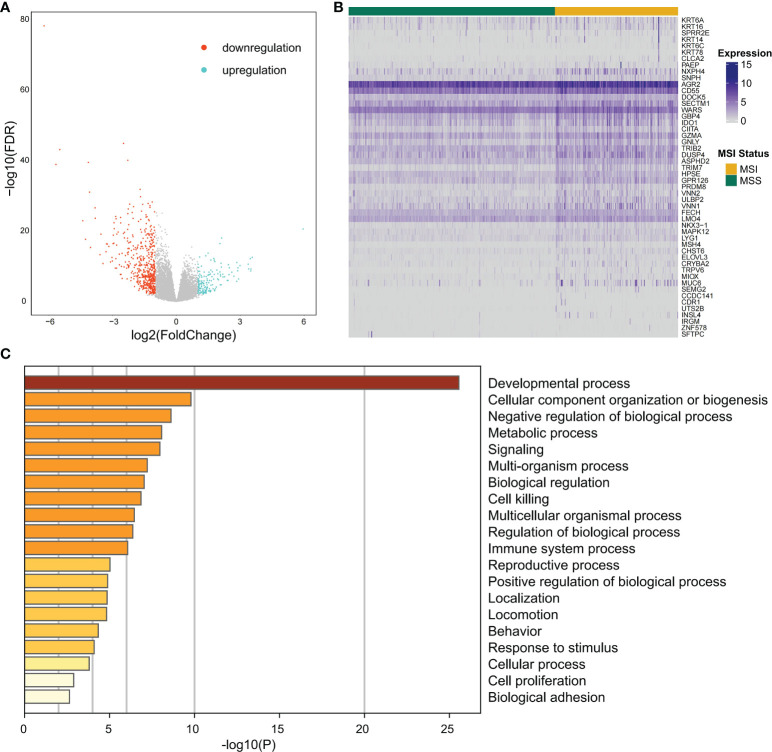
The differentially expressed gene identification and functional analysis. **(A)** Volcano plot for differential expression genes (DEGs) between MSI and MSS samples from TCGA COAD cohort. Blue points mean upregulation, and orange points mean downregulation. **(B)** The heatmap shows the expression of the top 50 DEGs in samples from TCGA COAD cohort. **(C)** For 701 significant DEGs, pathway and process enrichment analysis has been done with GO Biological Processes. The graphical graph revealed the top 20 enrichments having P < 0.01. A P-value was multi-test adjusted in log 10.

Differential expression analysis of the MSI and MSS samples was performed to identify genes that have a pivotal function in the microsatellite stability status, identifying 701 genes (**Figures 1**, **3A**), the top 50 of which are shown in the heatmap (**Figure 3B**). Pathway and process enrichment analyses using Metascape were used to detect the functional processes regulated by these 701 DEGs. Significantly enriched in GO biological processes were “Cellular component organization or biogenesis”, “Negative regulation of biological process”, “Developmental process”, “Metabolic process”, and so on (**Figure 3C**).

### Weighted gene co-expression network construction and immune-related modules identification

**Figure 4:**
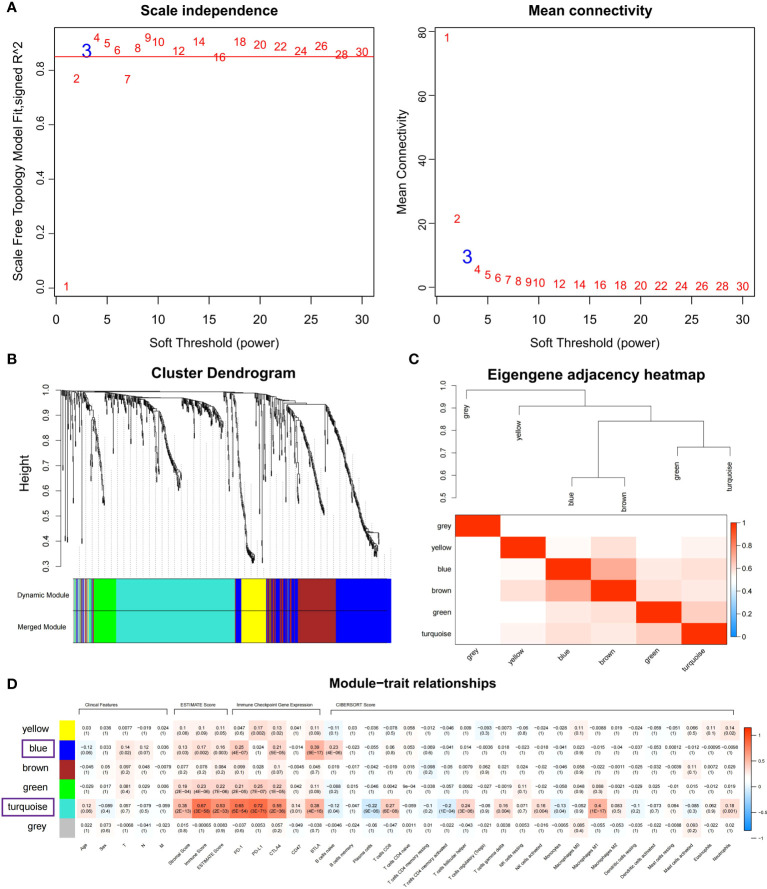
Identifying immune-related modules by WGCNA. **(A)** The scale-free fit index analysis and the mean connectivity for various soft-thresholding powers (β). **(B)** Dendrogram for clustering all differentially expressed genes relies on a measure of dissimilarity (1-TOM). **(C)** Clustering correlations among WGCNA modules. The color red represents a positive correlation, and blue represents a negative correlation. **(D)** Heatmap revealing the relationship between modules, clinical features, and immune factors, including ESTIMATE score, the immune checkpoints expression, and CIBERSORT 22 immune cell score. The red refers to a positive correlation, while the blue indicates a negative correlation.

Co-expressed networks were built by WGCNA according to 701 DEGs expressions in TCGA COAD cohort to identify the co-expression modules associated with immune traits (**Figure 1**). The module power value between 1 and 30 was evaluated to assure the average connectivity and high independence. To ensure a scale-free network, the power value was set to 3 when the scale-free R2 reached 0.9 as the soft-thresholding parameter (**Figure 4A**). The number of genes in each of the six modules identified was as follows: 179 in blue, 273 in turquoise, 122 in brown, 52 in green, 18 in gray, and 57 in the yellow module. The cluster tree is displayed in **Figure 4B**. The blue module was positively linked to brown and turquoise modules, and the turquoise module was positively correlated with green and blue modules (**Figure 4C**).

The module-trait association method was applied to detect the high co-expression modules relevant to the immune factors but did not affect clinical features. After gene clustering, the correlation between modules and phenotype was illustrated by heatmaps (**Figure 4D**). According to correlation analysis, blue and turquoise modules were identified as the immune-related modules highly correlated with ESTIMATE score (**Figures S2A, B**; Cor = 0.6, P = 4.4E-28 for turquoise; Cor = 0.15, P = 4.5E-02 for blue), the expression of PD-1 (**Figures S2C, D**; Cor = 0.72, P = 6.8E-45 for turquoise; Cor = 0.63, P = 3.5E-21 for blue) and BTLA (**Figures S2E, F**; Cor = 0.53, P = 3.6E-21 for turquoise; Cor = 0.85, P = 3.8E-51 for blue). Besides, the turquoise modules were highly linked to CD8 T cells (**Figure S2G**; Cor = 0.58, P = 6.1E-26) and M1 macrophage (**Figure S2H**; Cor = 0.6, P = 4.4E-28) infiltration. The co-expression network of blue and turquoise modules is displayed in **Figure S3**.

### The MSI-related prognostic model construction

**Figure 5:**
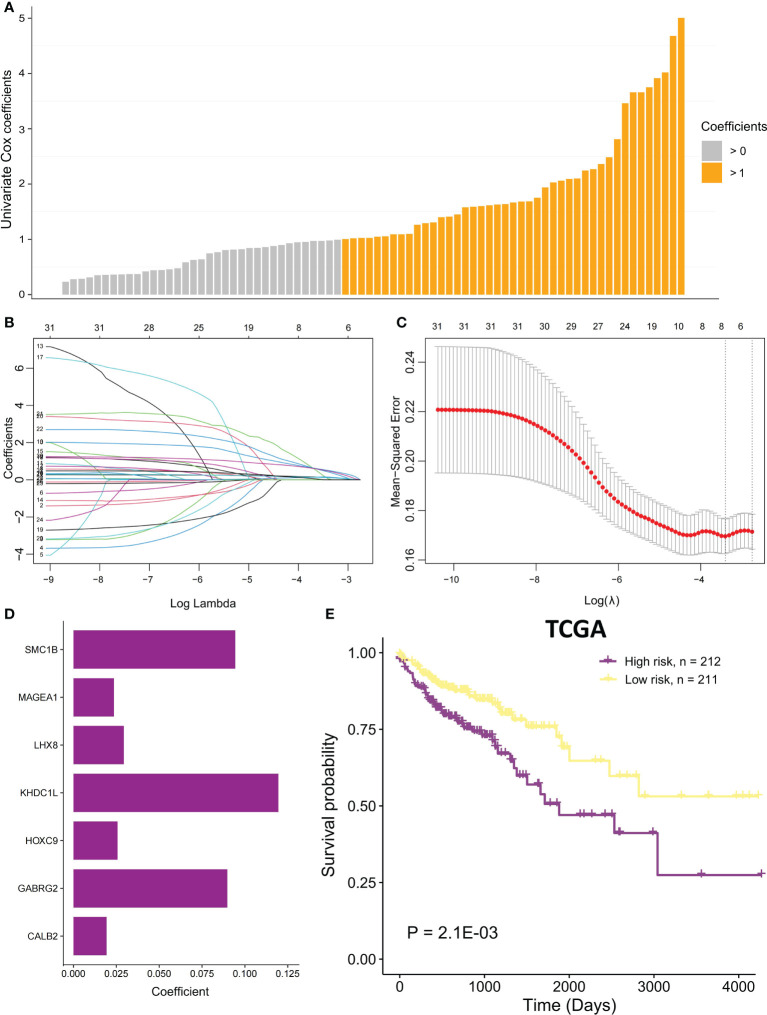
The MSI-related prognostic model construction. **(A)** Univariate Cox proportional regression analysis was conducted to identify significant MSI-related co-expression prognostic genes with P < 0.01. The bars mean coefficients of univariate Cox proportional regression analysis. **(B)** LASSO coefficient profiles of 78 MSI-related co-expression prognostic genes. **(C)** Cross-validation for tuning parameter selection in LASSO model. **(D)** The coefficients of seven MSI-related co-expression prognostic genes in the predictive model were caluclated. **(E)** In TCGA COAD cohort, OS difference among the two risk samples was evaluated using a log-rank test. Samples of high risk group: 212. Samples of low risk group: 211.

Univariate Cox proportional regression analysis was conducted to determine the prognostic value of selected MSI-related co-expression module genes, displaying that 78 MSI-related co-expression genes were statistically considerably linked to the overall survival (OS) (**Figures 1**, **5A**; P < 0.01). Next, LASSO analysis was utilized to identify the most effective prognostic genes in addition to one SE over the minimum threshold selected, leading to a model having seven MSI-related co-expression prognostic genes: SMC1B, MAGEA1, LHX8, KHDC1L, HOXC9, GABRG2, and CALB2 (**Figures 5B, C** **)**. Next, a predictive model was developed according to TCGA training set: risk score = (0.09433 * SMC1B expression) + (0.02362 * MAGEA1 expression) + (0.02937 * LHX8 expression) + (0.1195 * KHDC1L expression) + (0.02567 * HOXC9 expression) + (0.08978 * GABRG2 expression) + (0.01932 * CALB2 expression) (**Figure 5D**). In TCGA training set, every patient’s risk score was determined per the previous formula. The patients were categorized per the median risk score as the cutoff value into two risk groups, with the high-risk group having considerably poorer OS (**Figure 5E**; P = 2.1E-03; log-rank test).

**Figure 6:**
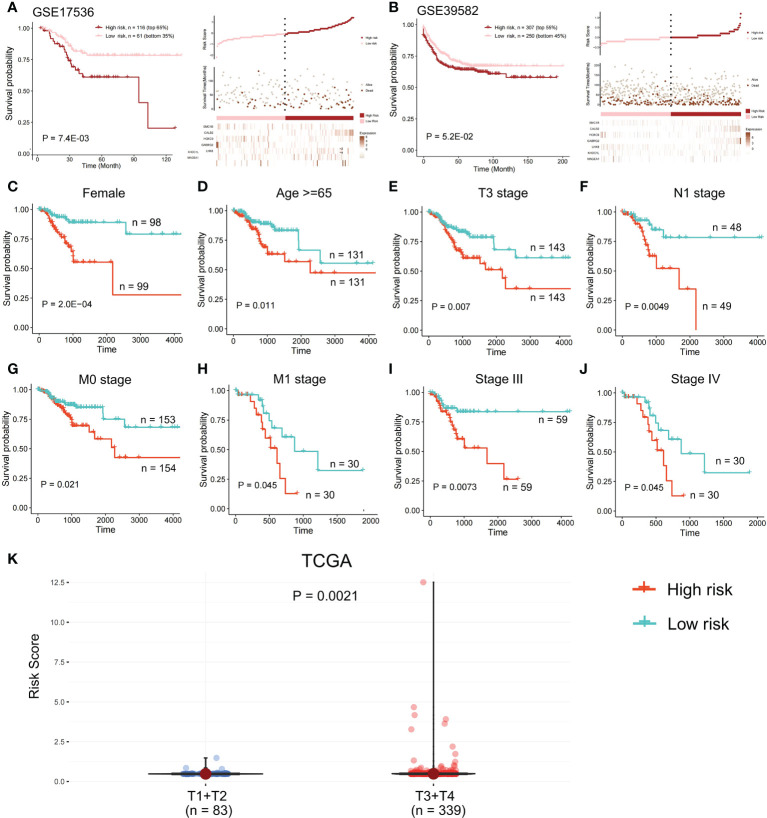
Survival analysis in the validation set. OS difference and the survival and risk score distribution among the two risk samples were evaluated using a log-rank test in **(A)** validation set GSE17536 and **(B)** validation set GSE39582. Samples of high risk group in GSE17536: 116. Samples of low risk group in GSE17536: 61. Samples of high risk group in GSE39582: 307. Samples of low risk group in GSE39582: 250. **(C-J)** In TCGA cohort, a log-rank test was employed to evaluate OS difference among two risk samples of females, older patients, T3 stage, N1 stage, M subgroups (M0 and M1), and pathological stage (Stages III and IV). **(K)** Risk score of T1+T2 group and T3+T4 group was compared.

**Figure 7:**
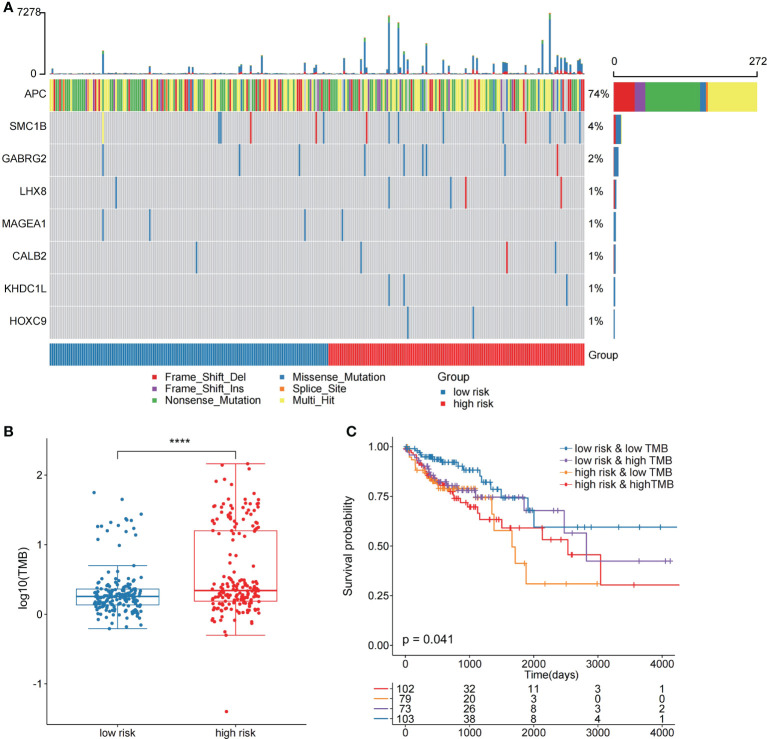
The two risk groups have different mutation features. **(A)** Somatic mutation features of the two risk groups. **(B)** TMB was compared between the two risk groups **(C)** Comparison of patient outcome of low-risk and low TMB group, low-risk and high TMB group, high-risk and low TMB group, and high-risk and high TMB group.

In the validation set GSE17536, the survival analysis revealed that the high-risk group had a poorer prognosis in OS (**Figures 6A**; P = 7.4E-03; log-rank test) and disease-specific survival (DFS) (**Figure S4**; P = 4.2E-02; logrank test), and more patients survived in the low risk group, whereas in the validation set GSE39582, the high-risk group had a poorer prognosis (**Figures 6B**; P = 5.2E-02; log-rank test), and more patients survived in the low-risk group. Further investigations were performed to confirm if the risk score indicates prognosis for distinct subgroups of clinical characteristics. In TCGA cohort, females, older patients, T3 stage, N1 stage, pathological stage (Stages III and IV), and M subgroups (M0 and M1), the high-risk group patients, presented a considerably poorer OS (**Figures 6C–J**; P < 0.05; log-rank test). We also found that the risk score of T3+T4 group was higher than that of T1+T2 group (**Figure 6K**). APC gene had the most mutations in COAD (**Figure 7A**) and TMB is higher in the high-risk group (**Figure 7B**, Wilcoxon test, P<0.0001). During the comparison of patient prognosis of low risk and low TMB group, low risk and high TMB group, high risk and low TMB group, and high risk and high TMB group, the patients of the four groups had different outcomes (**Figure 7C**; P=0.041).

Then, we compared the two risk groups’ genetic mutation status. In TCGA cohort, **Figures S5A, B** revealed the top 20 mutations in the two risk samples. The top five mutations and prognosis correlation were analyzed in the high-risk group, showing that KARS mutation was linked to a poor prognosis (**Figure S5C**; P = 0.072). However, no difference was observed between the low-risk group and the entire TCGA COAD cohort (**Figures S5D, E**).

### Risk scores were related to immune features and microsatellite stability status

**Figure 8:**
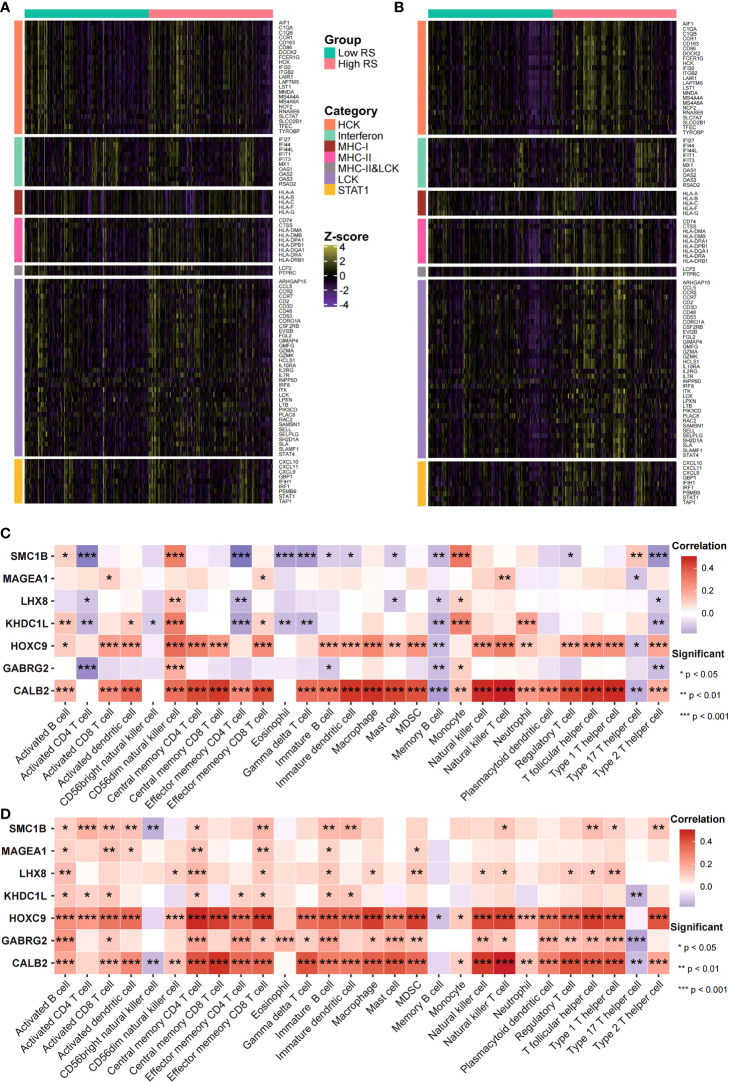
Risk scores were related to cancer’s immune features. **(A, B)** Expression status of immune-related genes in GEO **(A)** and TCGA **(B)** data sets was analyzed. **(C, D)** The risk score and immune infiltration level correlation in GEO **(C)** as well as TCGA **(D)** data sets were analyzed.

**Figure 9:**
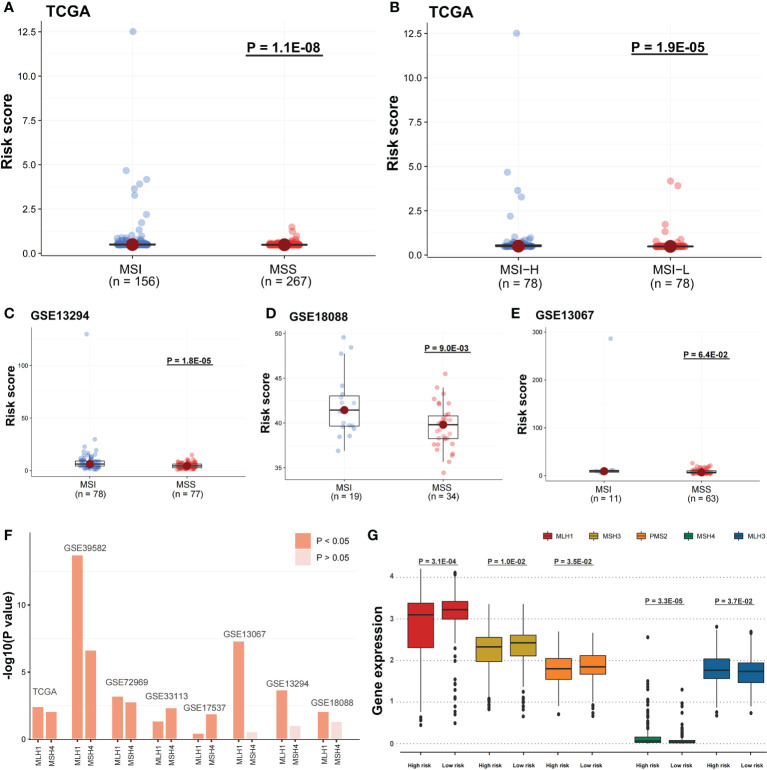
Risk scores were related to microsatellite stability status and the MMR gene expression. **(A)** A one-sided Wilcoxon rank-sum test was utilized for evaluating risk score differences in TCGA cohort between MSI and MSS groups and **(B)** MSI-H and MSI-L groups, and **(C-E)** in GSE13294, GSE18088, and GSE13067 cohorts between MSI and MSS groups. **(F)** Pearson correlation analysis was performed to evaluate the correlation between DNA mismatch repair (MMR) gene expression and risk score. The bars mean -log10 (P-value). **(G)** In TCGA cohort, the five MMR gene expression differences among the two risk groups were evaluated by a one-sided Wilcoxon rank-sum test. Statistical significance is determined when P-value < 0.05.

The risk score potential in predicting COAD’s immune features was determined by first illustrating the expression status of immune-related genes in the two risk groups of the two data sets (**Figures 8A, B**). The analysis of the linkage between the immune cell infiltration and expression levels of risk score component genes indicated that HOXC9 and CALB2 are significantly correlated with most immune cell infiltration levels (**Figures 8C, D**). To confirm the associations between risk score and microsatellite stability status, we analyzed the risk score difference of different microsatellite stability statuses (**Figure 1**). In TCGA COAD cohort, MSI group had a higher risk score than MSS group (**Figure 9A**; P = 1.1E-08; one-sided Wilcoxon rank-sum test), and the risk score in MSI-L group was lower than MSI-H group (**Figure 9B**; P = 1.9E-05; one-sided Wilcoxon rank-sum test). Besides, the risk score in MSI group was higher than MSS group in GSE13294 (**Figure 9C**; P = 1.8E-05; one-sided Wilcoxon rank-sum test), GSE18088 (**Figure 9D**; P = 9.0E-03; one-sided Wilcoxon rank-sum test) and GSE13067 (**Figure 9E**; P = 6.4E-02; one-sided Wilcoxon rank-sum test) cohorts. Next, Pearson correlation analysis was conducted to analyze the correlation between the risk score and expression level of MLH1 and MSH4 (**Figure 9F**). MutS homologues are the major conductor of the correction of errors introduced in microsatellites. MLH1, MSH3, PMS2, MSH4, MLH3 are five component genes of MutS homologues which can recognize mismatched nucleotides to initiate the repair process (24, 25). Thus, here we analyzed the differential expression status of the five genes in high risk score group and low risk score group. Differences in the five MMR gene expression levels in the high-risk and low-risk score groups are presented in **Figure 9G**.

### The risk score and drug response correlation

**Figure 10:**
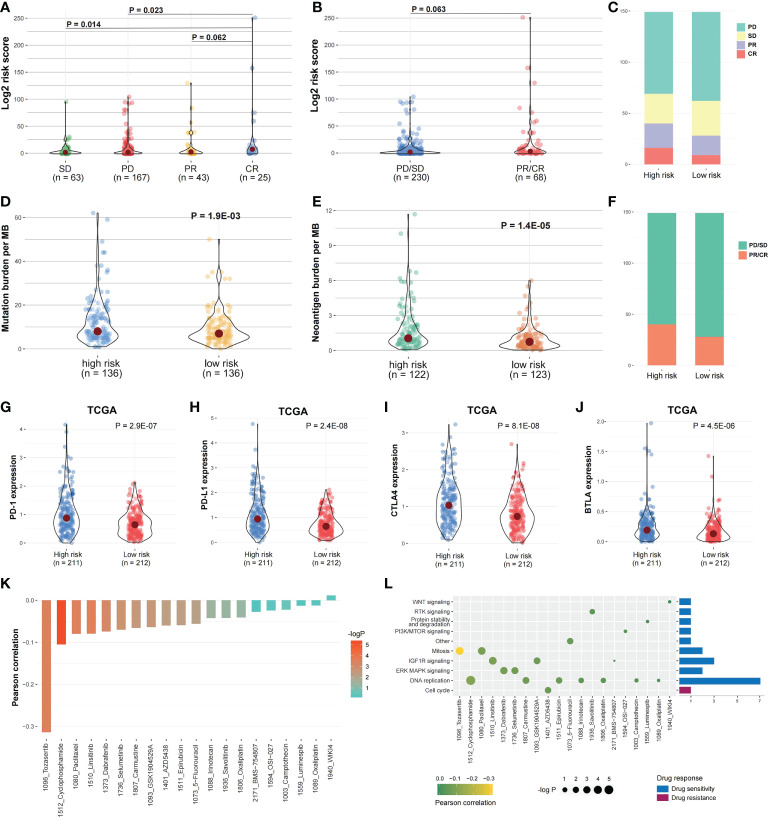
Risk score and drug response correlation. **(A, B)** In IMvigor210 cohort, the risk score distribution between the responsive groups and **(C, F)** between the two risk groups. **(D, E)** The mutation and Neoantigen burden distribution in the two risk group patients. **(G–J)** The immune checkpoints expression among the two risk groups. **(K)** The risk score and drug response value correlation using Pearson correlation analysis. Each column refers to a drug. Column brightness represents correlation significance. The column height represents a correlation. **(L)** Signaling pathways targeted by drug resistance to the risk score or sensitivity are presented in red and blue, respectively. Drug names and the signaling pathway targeted by the drug are presented on horizontal and vertical axes, respectively. The number of drugs targeting every signaling pathway is shown on the right of the bar graph. The point size represents the correlation significance.

In IMvigor210 cohort, the risk score differences among the immunotherapy responsive groups were evaluated to determine if the risk score can predict patients’ immunotherapy response (**Figure 1**). The risk scores in SD and PD were significantly higher than in CR (**Figure 10A**; P < 0.05), while the risk score in PR was higher compared to CR (**Figure 10A**; P = 0.062). The immunotherapy responsive group risk score was higher compared to non-response group (**Figure 10B**; P = 0.063). The high-risk patients with PD or SD responses were less than low-risk patients, and the high-risk group patients with PR or CR responses were more than low-risk patients (**Figures 10C, F** **)**. Besides, the mutation and neoantigen burdens in high-risk patients were higher (**Figures 10D**, **E**; P < 0.05). Taken together, such findings indicate that high-risk patients showed better immunotherapy response in IMvigor210 cohort. Then, we investigated the expression of the immune checkpoints among the two risk groups, with the high-risk group in TCGA COAD cohort having considerably higher PD-1, PD-L1, BTLA, and CTLA4 (**Figures 10G-J**; P < 0.05). In IMvigor210 cohort, the high-risk group had higher PD-1 as well as CD47 (**Figure S6**).

We then examined the linkage between risk score and responsiveness to 20 antitumor agents in GDSC cancer cell lines. Nineteen drugs with a drug response value (LN_IC50) were negatively linked to the risk score, defined as “drug sensitivity”, whereas one drug was positively linked, defined as “drug resistance”, by Pearson correlation analysis (**Figure 10K**). The drugs with sensitivity were mostly targeting DNA replication and IGF1R signaling pathways (**Figure 10L**).

### Immunohistochemical pathological analysis

**Figure 11:**
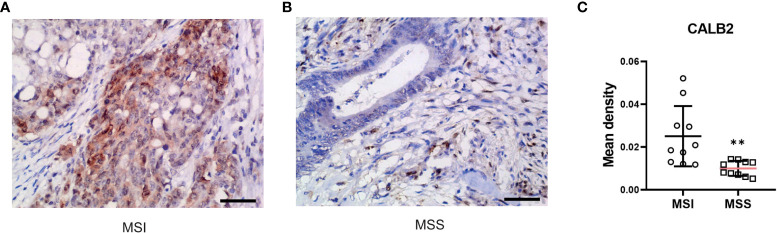
CALB2’s expression status in clinic acquired tissues. Representative images of IHC-stained MSI COAD tissue **(A)** and MSS COAD tissue **(B)**. **(C)** Comparison of immunohistochemical expression of CALB2 in MSI COAD and MSS COAD tissues. N=20.

To further validate the prognostic value of identified core genes, immunohistochemical pathological analysis was executed to analyze gene CALB2 protein expression status in MSI and MSS subtypes, demonstrating that the gene had a higher expression level in MSI samples (**Figures 11A-C**).

## Discussion

COAD treatment is challenging because of the advanced stage and poor OS; accordingly, new therapeutic targets are necessary (26). MSI is a high-frequency event in CRC, and recent studies have revealed that MMR deficiency/MSI-H status affects the response to ICI treatment in mCRC patients (27, 28). However, the efficacy of MSI for COAD treatment requires further research. Currently, genes are used to establish a predictive model to evaluate COAD prognosis and responsiveness to therapy, and several gene signatures have been constructed using large-scale publicly available datasets (29, 30). Consequently, the current study established an MSI and immune-related prognostic model comprising seven genes to identify COAD patients who may have better immunotherapy responsiveness. We further validated OS predicting the efficacy of this model in COAD patients *via* a validation analysis for prognostic signatures. The prognostic model can distinguish COAD patients with different responses to ICI treatment.

As an assessment of microsatellite status, we considered the order of “MSS MSI-L MSI-H” as progressive relationships, revealing that the infiltration of “M1 macrophages”, “follicular helper T cells”, and “neutrophils” in MSS samples was significantly lower compared to MSI samples; a similar pattern was observed in the comparison of MSI-H and MSI-L (P < 0.05). M1 macrophages are activated macrophages, defined due to pro-inflammatory cytokine production, mediating pathogens resistance, and exhibiting strong microbicidal characteristics (31). In addition, they are tissue destructive and have anti-tumoral ability (32). Follicular helper T cells are a subpopulation of CD4^+^ T cells that have a pivotal function in protective immunity because they assist B cells in antibody production versus foreign pathogens (33). Neutrophils have a pivotal function in the host defense versus infection (34). Meanwhile, high T cell infiltration is linked to a favorable cancer prognosis (8, 35). In addition, the immune score was assessed according to ESTIMATE algorithm, and the five immune checkpoints expression increased as MSI level increased (P < 0.05). These findings indicate that MSI is linked to the proportion of immune cell infiltration in COAD; higher microsatellite stability indicates an increase in immune infiltration.

This study identified DEGs related to the microsatellite instability status and functional analysis disclosed that DEGs were significantly enriched in several regulatory pathways. The term “Developmental process” refers to some biological changes linked to growth, information transfer, and differentiation over the organism’s life cycle. “Cellular component organization or biogenesis” leads to the constituent parts assembly or a cellular component disassembly. “Negative regulation of biological process” represents any process that reduces, prevents, or stops the biological process rate, frequency, or extent. “Metabolic processes” are chemical reactions and pathways, such as catabolism and anabolism. Our analysis revealed that DEGs related to the microsatellite stability status were closely associated with the growth and activity of cellular components and organisms.

A prognostic model was constructed of seven MSI and immune-related genes in COAD according to WGCNA and other bioinformatics analyses. This approach has demonstrated its effectiveness in cancer research and is commonly utilized (36). The biomarkers identified also have a stable efficacy in COAD prognosis. Apart from T stage, M stage, N stage, Stage and age, gender and sex hormone also contribute to disease prognosis of COAD (37). To further validate our score’s robustness, we analyzed the prognosis of high risk patients and low risk patients in subgroups with different clinical pathology features. Results further demonstrated our risk score’s effectiveness. SMC1B associates with cohesin proteins and plays a part in genome stability (38). MAGEA1 codes for an antigen that may cause cancer immune suppression (39). LHX8 is a crucial transcription factor mostly expressed in germ cells (40). HOXC9 controls various cellular processes linked to differentiation *via* activating and repressing the transcription of different gene sets (41). Mutations in GABRG2 have been associated with epilepsy syndromes with varying severities (42). CALB2 is expressed in most poorly differentiated colon carcinomas (43). Although the function of KHDC1L is unclear, we still approve of its effect in the prognostic model. Our prognostic model can differentiate high and low-risk patients, not only in TCGA COAD cohorts (even clinicopathological subgroups) but in the validation sets GSE17536 and GSE39582. Moreover, there were considerable risk scores differences between MSI and MSS samples in TCGA, GSE13294, GSE18088, and GSE13067 datasets, signifying that the prognostic model reflects the microsatellite stability status of patients.

The immunotherapy dataset IMvigor210 was used to validate our prognostic model. Although the cancer type of patients in IMvigor210 is mUCC, IMvigor210 was widely used as a validation dataset in some studies about other cancers, such as glioblastoma and hepatocellular carcinoma (44, 45). The high-risk patients’ score was more likely to profit from ICI (atezolizumab, an anti-PD-L1 antibody) treatment. In addition, we observed a considerable upregulation of immune checkpoints expression in TCGA and IMvigor210, especially PD-L1. The findings revealed that our predictive model might identify groups more susceptible to immunotherapy, and it has potential predictive power for other cancer types (46).

Next, to further validate the potential of our research in clinic applications, we conducted an immunohistochemical pathological analysis. CALB2 (Calbindin 2) encodes an intracellular calcium-binding protein belonging to the troponin C superfamily. This protein plays an important role in message targeting and intracellular calcium buffering and related to cancer progression (47–49). Among DEGs of MSI-H and MSI-L subtypes, CALB2 has the highest fold change. Furthermore, recent research revealed its prognostic value in predicting the outcome and therapy resistance of COAD patients (43, 50). However, the difference in CALB2 expression status in MSI and MSS subtypes remains unclear; consequently, further analysis of clinic-acquired COAD tissues was performed, displaying that CALB2 has a higher expression level in MSI samples which further indicated the therapeutic potential of CALB2.

In conclusion, through a sequence of bioinformatics analyses, a seven-gene predictive model was created to predict COAD patients’ outcomes. It could accurately distinguish COAD patients with different prognoses. By categorizing patients and determining a suitable therapy course, our data may help choose the precision medicine in COAD.

## Data availability statement

The raw data supporting the conclusions of this article will be made available by the authors, without undue reservation.

## Ethics statement

The present study was approved by the ethics committee of the Fourth Affiliated Hospital of Harbin Medical University. The patients/participants provided their written informed consent to participate in this study.

## Author contributions

ML, ZS, GL, and DS contribute to conception and design of the study. ZS, GL, and DS wrote or contributed to the writing of the manuscript. ZS, JZ, and LA collected the data. ZS, DS, and JZ conducted the bioinformatic analysis. ML contributed to the material support of the study. All authors contributed to the article and approved the submitted version.

## Funding

This work was supported by the National Natural Science Foundation of China (Grant No. 82072673) and the Natural Science Foundation of Heilongjiang Province of China (Grant No. LH2020H066).

## Conflict of interest

The authors declare that the research was conducted in the absence of any commercial or financial relationships that could be construed as a potential conflict of interest.

## Publisher’s note

All claims expressed in this article are solely those of the authors and do not necessarily represent those of their affiliated organizations, or those of the publisher, the editors and the reviewers. Any product that may be evaluated in this article, or claim that may be made by its manufacturer, is not guaranteed or endorsed by the publisher.

## Abbreviations

COAD, Colon adenocarcinoma; MSI, Microsatellite instability; MSS, Microsatellite stability; CRC, Colorectal cancer; dMMR, Mismatch-repair-deficiency; MSI-H, High levels of microsatellite instability; pMMR, Mismatch-repair-proficient; ICI, Immune checkpoint inhibitor; TME, Tumor microenvironment; TCGA, The Cancer Genome Atlas; GEO, Gene Expression Omnibus; MSI-L, Low levels of microsatellite instability; IOD, Immunohistochemical integral optical density; OS, Overall survival
