# Supplementary material for: Identification of microsatellite instability and immune-related prognostic biomarkers in colon adenocarcinoma
Source: Front Immunol. 2022 Oct 7;13:988303. doi: 10.3389/fimmu.2022.988303 (PMC9585257; doi:10.3389/fimmu.2022.988303)
Supplement: Supplementary file 4 [file DataSheet_1.pdf]

Supplementary Information

Supplementary Tables

Table S1. 701 DEGs between MSI and MSS

Table S2. Pearson correlation analysis of modules and traits

Table S3. 22 immune cells and signatures

Supplementary Figures

Figure S1. Infiltration of T cells CD8 between different microsatellite stability status

Figure S2. The correlation between blue and turquoise modules genes and immune traits

Figure S3. The co-expression network of blue and turquoise modules

Figure S4. Survival analysis in validation set GSE17536

Figure S5. High risk and low-risk groups with different distribution of mutation

Figure S6. The expression of immune checkpoints between high and low risk group

Supplementary Figures

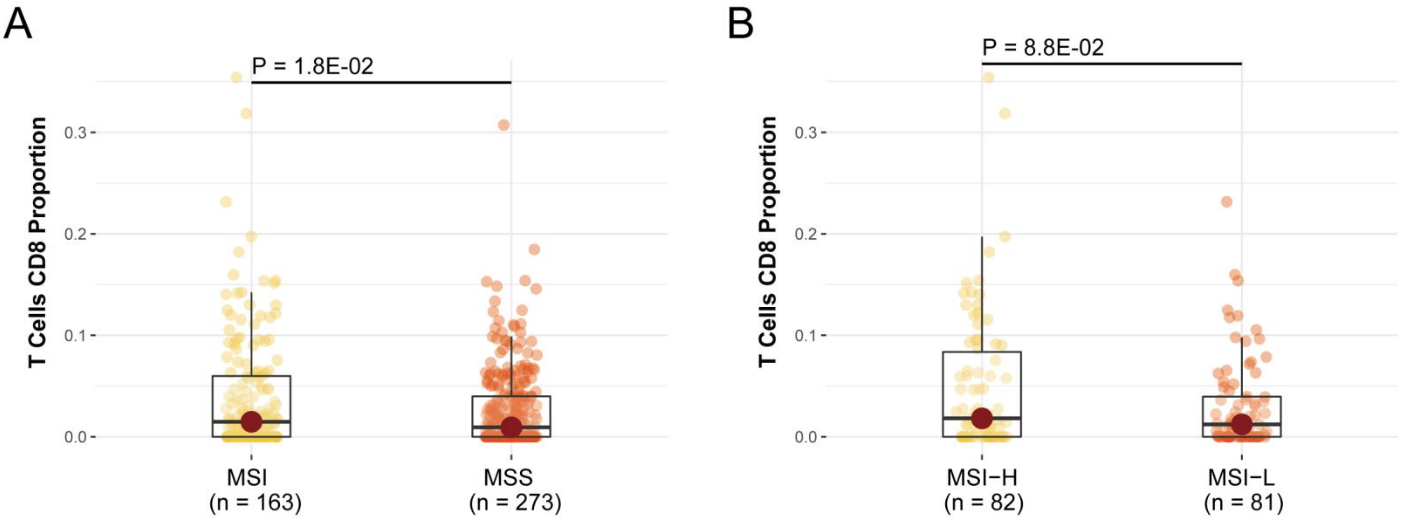

Figure S1. Infiltration of T cells CD8 between different microsatellite stability status

(A) Infiltration of T cells CD8 between MSI group and MSS group. **(B)** Infiltration of T cells CD8 between MSI-H group and MSI-L group.

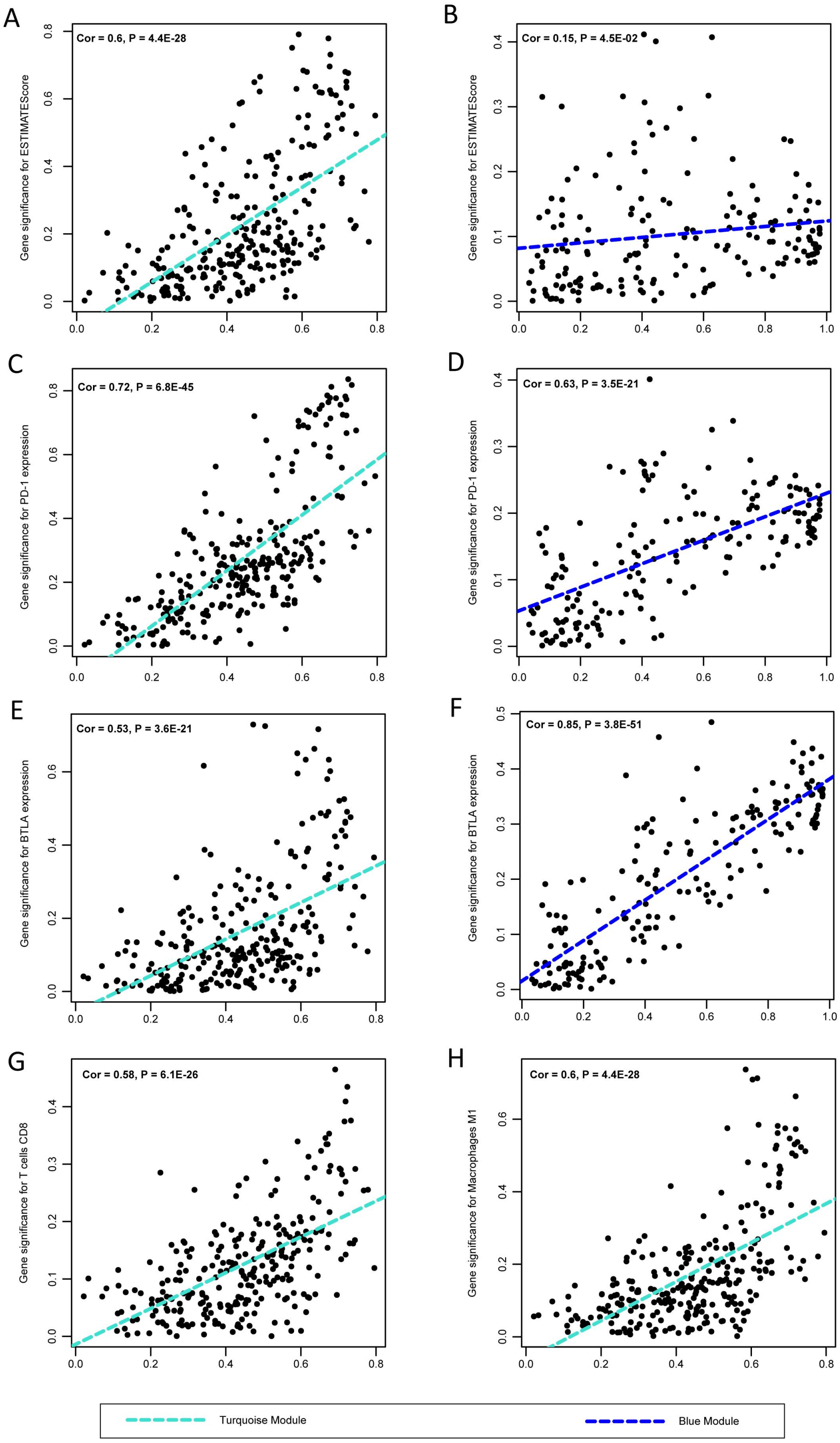

Figure S2. The correlation between blue and turquoise modules genes and immune traits

(A)-(B) The correlation of blue and turquoise modules with ESTIMATE scores. **(C)-(D)** The correlation of blue and turquoise modules with the expression of PD-1. **(E)-(F)** The correlation of blue and turquoise modules with the expression of CTLA4. **(G)-(H)** The correlation of turquoise module with T cells CD8 and Macrophages M1 infiltration.

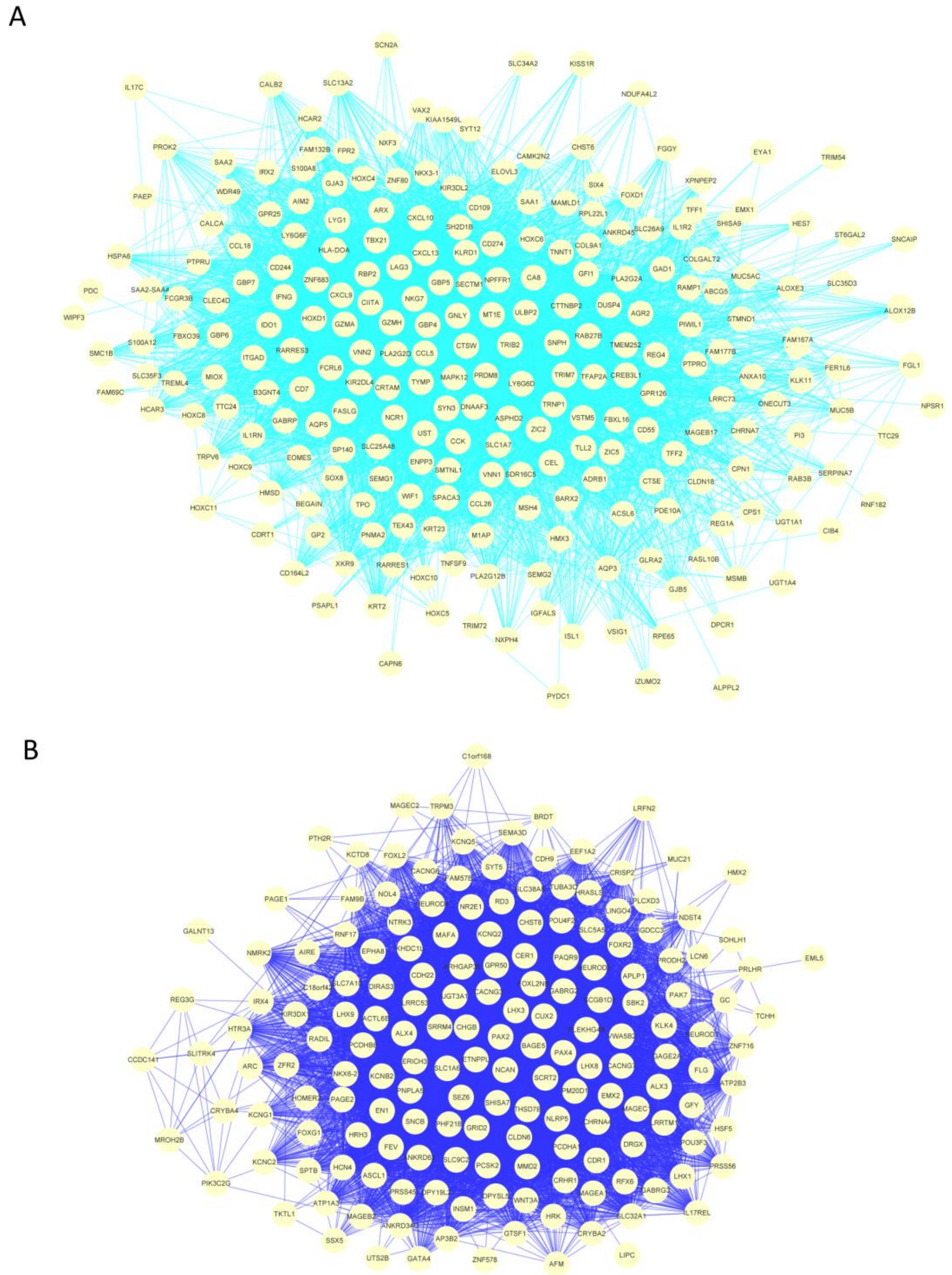

Figure S3. The co-expression network of blue and turquoise modules

(A) The co-expression network of blue modules. **(B)** The co-expression network of turquoise modules. The nodes mean genes. The width of edges represents the degree of correlation, and the wider the edge, the stronger the correlation of genes.

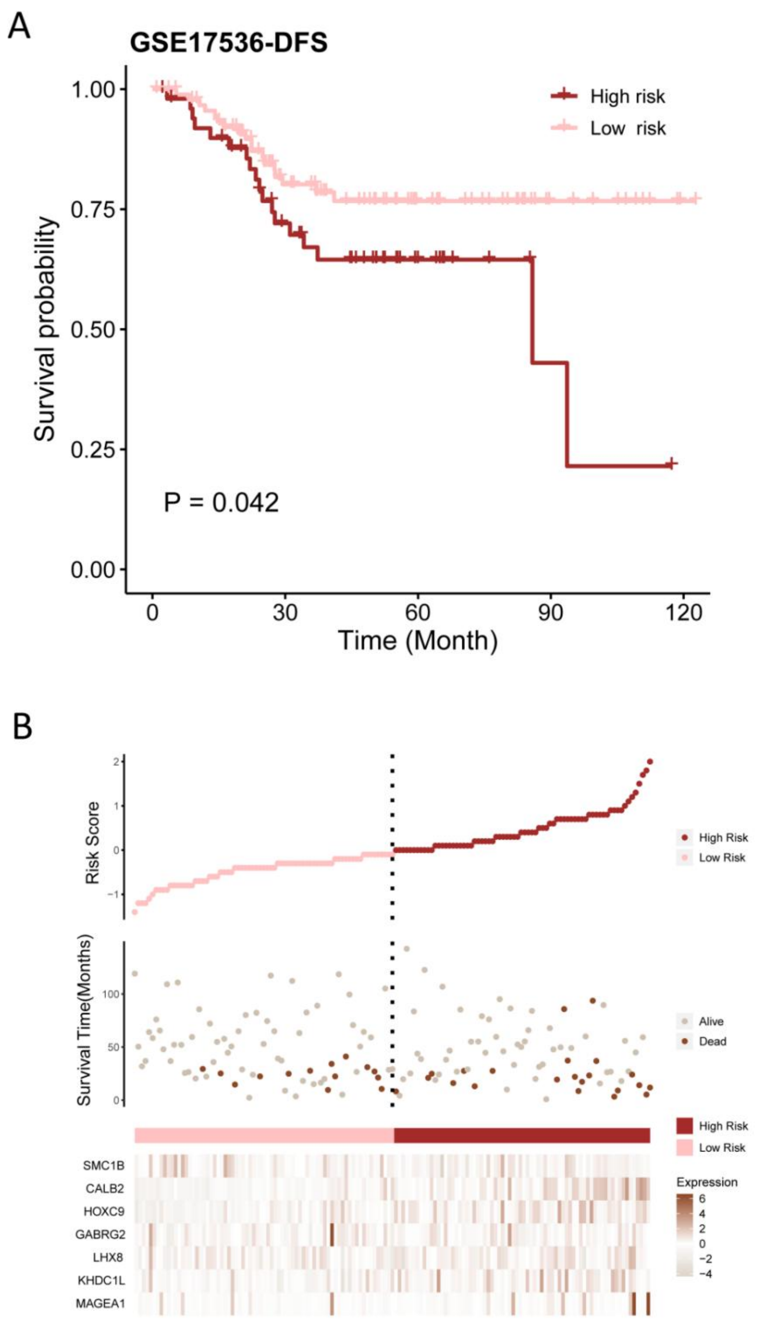

Figure S4. Survival analysis in validation set GSE17536

(A) Log-rank test was used to assess the difference in disease specific survival (DFS) between high risk and low risk samples in validation set GSE17536. **(B)** Distribution of survival time and risk score which was calculated based on the expression of seven MSI-related co-expression prognostic genes in validation set GSE17536.

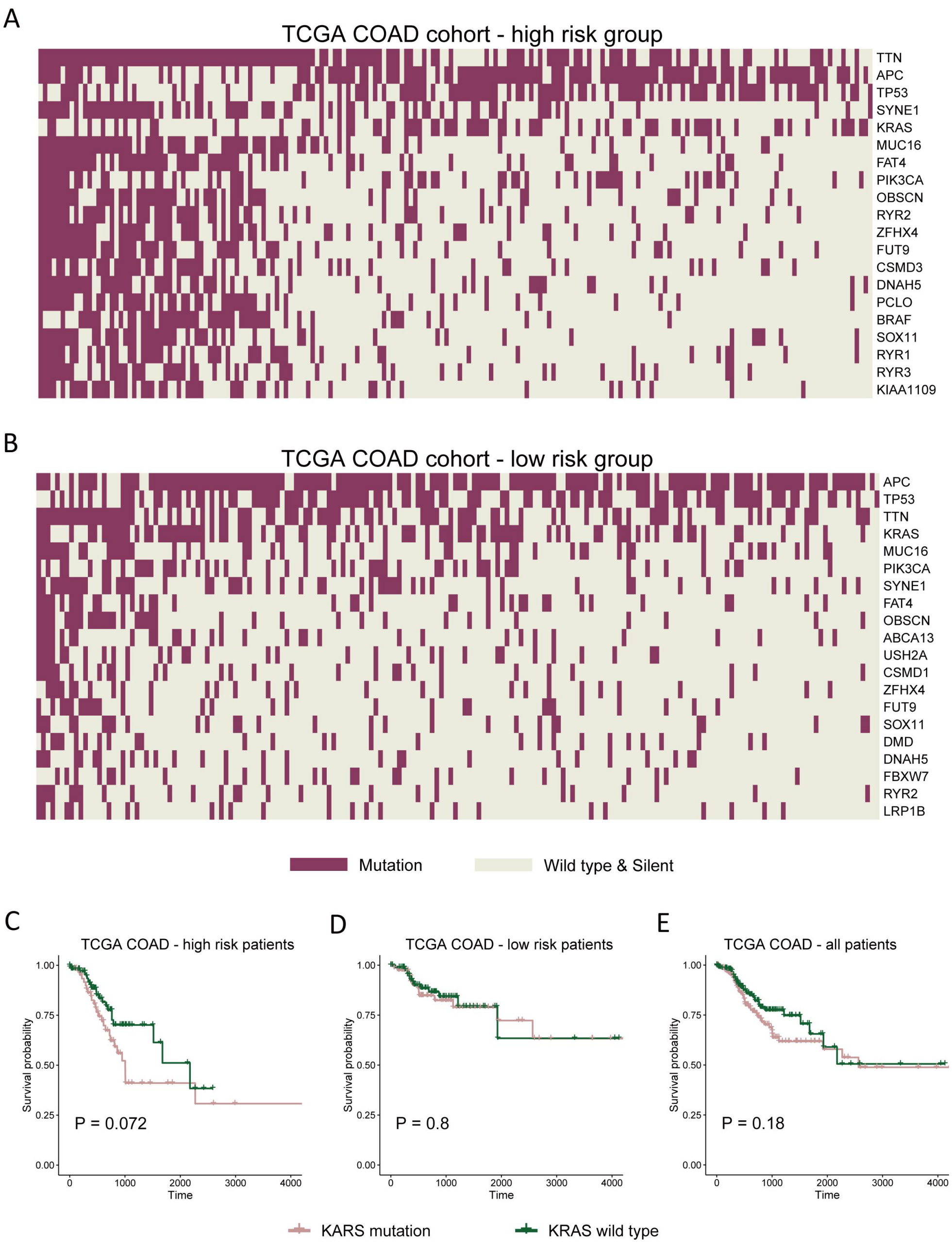

Figure S5. High risk and low-risk groups with different distribution of mutation

(A) The distribution of top 20 genes with high mutation frequency in high risk group of TCGA COAD cohort. **(B)** The distribution of top 20 genes with high mutation frequency in low risk group of TCGA COAD cohort. **(C)-(E)** Log-rank test was used to assess the difference in OS between KRAS mutation and wild type samples in COAD cohort with high risk, COAD cohort with low risk, and TCGA COAD cohort.

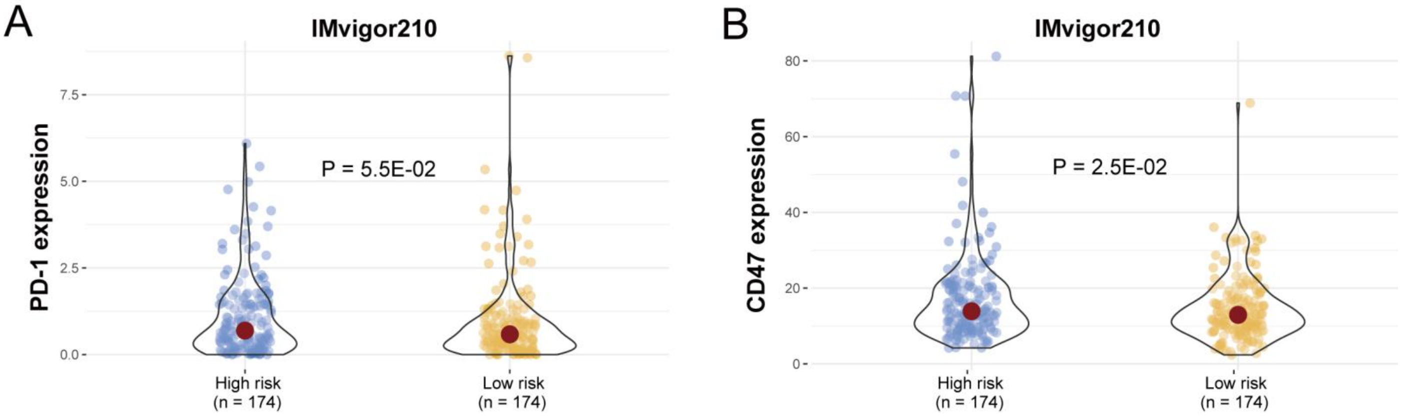

Figure S6. The expression of immune checkpoints between high and low risk groups

(A) The expression of PD-1 between high risk group and low risk groups in IMvigor210 cohort. **(B)** The expression of CD47 between high risk group and low risk groups in IMvigor210 cohort.
